# Parametric mapping of dynamic ^68^Ga FAPI-46-PET data of 42 patients with pancreatic lesions

**DOI:** 10.1186/s40644-026-01040-w

**Published:** 2026-04-28

**Authors:** Anna-Maria Spektor, Isabelle von Goetze, Hans-Georg Buchholz, Ulrike Heger, Matthias Lang, Jakob Liermann, Maximilian Knoll, Klaus Herfarth, Mathias Schreckenberger, Jürgen Debus, Uwe Haberkorn, Manuel Röhrich

**Affiliations:** 1 Department of Nuclear Medicine, University Medical Center Mainz, Langenbeckstraße 1, 55131 Mainz, Germany; 2 Department of Surgery, University Hospital Heidelberg, Heidelberg, Germany; 3 Department of Radiation Oncology, University Hospital Heidelberg, Heidelberg, Germany; 4 Department of Nuclear Medicine, University Hospital Heidelberg, Heidelberg, Germany

**Keywords:** Fibroblast activation protein, FAPI, PET, Cancer, PDAC

## Abstract

**Purpose:**

Differential diagnoses of primary pancreatic lesions include pancreatic ductal adenocarcinomas (PDAC) and inflammatory lesions of the pancreas (ILP). Post-pancreatic surgery, differentiation of postoperative reactive tissue (PRT) and PDAC-recurrence challenges oncological imaging. Static ^68^Ga-FAPI-PET/CT uptake is increased in all of these lesions with marked overlap in signal intensity, hampering their FAPI-PET-based assessment. Here, we evaluated static and parametric imaging parameters for discrimination of pancreatic lesions in primary and post-pancreatic surgery scenarios.

**Methods:**

55 Patients with pancreatic lesions (36 primary, 19 post-pancreatic surgery) underwent static and dynamic ^68^Ga-FAPI-46-PET/CT. Primary lesions were classified either by histology following PET/CT or follow-up (> 6 months). Post-surgery, PRT and PDAC-recurrence were classified by CT- and clinical course (> 18 months). Parametric maps (1 tissue compartment (1TC), 2TC and Logan plot (LP)) from dynamic PET-data were generated via image-based aortic input function using PMOD-software. Pancreatic lesions (PDAC, ILP, PRT, PDAC-recurrence) were then delineated using VOI-technique (30–70% isocontour) and signal intensities were analyzed. SPSS was used to detect outliers, unpaired t-tests was applied for comparison of static and parametric imaging parameters. Receiver-operating-characteristic curves for differentiating PDAC/ILP or recurrent PDAC/PRT were generated.

**Results:**

42 patients were included in the final analysis: in primary setting, 16 PDAC and 10 ILP; in post-surgery setting 9 PDAC-recurrences and 7 PRT. In the primary setting, although PDAC showed higher SUVmax/mean than ILP, no significant differences in maximum/mean signal values neither in static imaging nor in parametric maps were detected. With regard to the differentiation of PDAC-recurrences versus postoperative tissue, LPmax were significantly higher in PDAC-recurrences compared to PRT (4.74 vs. 2.40, p-value 0.020) with AUC 82.5% (95-CI 0.62-1.0) and a possible diagnostic threshold at > 3,49 (LR + 5.44), while differences in static imaging or other parametric maps were not statistically significant.

**Conclusion:**

Differentiating pancreatic lesions remains challenging. While LPmax significantly distinguished PDAC-recurrence from PRT, other parametric mapping parameters yielded no significant results. Larger studies and additional dynamic data analysis methods should be explored.

**Supplementary Information:**

The online version contains supplementary material available at 10.1186/s40644-026-01040-w.

## Introduction

Pancreatic ductal adenocarcinoma (PDAC) is the most common pancreatic lesion and a highly lethal tumor with a five-year survival rate of less than 12% due to late diagnosis and a high rate of recurrence [1–3]. Cystic neoplasms like intraductal papillary mucinous neoplasms (IPMN), PDAC recurrences, post-operative inflammation or primary inflammatory lesions of the pancreas (ILP) are potential differential diagnosis of PDAC when radiologically detected in a primary or follow-up setting [3]. Thus, early detection and differentiation between new primary pancreatic lesions, true tumor recurrence and benign inflammatory or reactive changes in primary and post-therapy imaging is crucial for guiding appropriate clinical management [4–6].

Primary pancreatic lesions are diagnosed with magnetic resonance imaging (MRI), computed tomography (CT) and endoscopic ultrasound (EUS). For differentiation of PDAC or ILP, neither CT, MRI nor ^18^F-fluorodeoxyglucose positron emission tomography/CT (^18^F-FDG PET/CT) showed promising results in previous studies [7–9]. Here, fibroblast activation protein inhibitor- (FAPI) PET/CT has emerged as a promising imaging modality for differentiation of benign and malign lesions [10–13] as FAP is overexpressed in cancer-associated fibroblasts (CAFs) in many stromal tumors, including PDAC, while showing minimal expression in normal tissues [14, 15]. Local PDAC-recurrence is diagnosed clinically by elevated CA19-9 levels and CT-morphological tissue growth in a follow-up setting. Nevertheless, the differentiation between local inflammatory changes and recurrence is still a diagnostic challenge [16–19]. Although ^18^F-fluorodeoxyglucose positron emission tomography/computed tomography (^18^F-FDG PET/CT) has become a widely used imaging modality for the evaluation of suspected cancer recurrence, its utility in distinguishing recurrent PDAC from post-treatment inflammatory lesions is limited by the fact that both can exhibit elevated FDG-uptake due to activated inflammatory cells leading to potential diagnostic pitfalls [20, 21]. Although studies have shown that FAPI-PET/CT is more specific than FDG-PET/CT in identifying and staging of PDAC [22, 23], static FAPI-PET/CT imaging faces challenges similar to FDG-PET/CT: inflammatory lesions, particularly those associated with chronic inflammation or fibrosis, can still exhibit FAP, leading to false positives in the assessment of tumor recurrence [24, 25].

To enhance the accuracy of FAPI-PET parametric imaging techniques have been explored in oncological and non-oncological setting using FDG, FET and a single time FAPI indicating a better tumor contrast and thus more precise diagnostics [26–31]. In glioma, for instance, better differentiation between recurring and non-recurring voxels was observed [31]. Parametric imaging involves the voxel-wise analysis of dynamic PET data, allowing the generation of images that reflect different kinetic parameters and behavior of the applied tracer [32]. This method allows the generation of images that reflect the rate of FAPI-uptake, binding potential and washout rates. As malignant lesions typically show different kinetic profiles in dynamic images compared to benign inflammatory processes [10, 30], we hypothesize that the specificity and sensitivity of the modality in distinguishing malignancy from inflammation may increase.

In this retrospective analysis, we evaluate parametric mapping derived from dynamic ^68^Ga-FAPI-46-PET data in order to assess the FAPI-uptake behavior of pancreatic lesions in primary and recurrence scenarios. The aim of this study is to analyze whether parametric imaging supports the differentiation of PDAC from ILP and PDAC-recurrence from benign post-surgery reactive tissue (PRT).

## Materials and methods

### Patient characteristics

Patients with primary pancreatic lesions or status post PDAC-surgery in a follow-up situation were selected in the period between September 2018 and September 2023 according to the following criteria: age over 18 years, CT- or MRI-proven lesion most consistent with PDAC or ILP in primary setting, PDAC-recurrence or PRT in follow-up setting; given indication for ^68^Ga-FAPI-PET/CT and biopsy/resection according to the local interdisciplinary tumor conference (ITC); sufficient compliance for and consent to the ^68^Ga-FAPI-PET/CT procedure and further data analysis; in cases without histology, MRI-, CT-, and laboratory follow-up data (> 6 months). Exclusion criteria for further analysis were other final histological diagnoses than PDAC, ILP, PRT or PDAC-recurrence, missing follow-up imaging or clinical data, and incomplete image data. All patients meeting these selection criteria were included.

### Dignity classification

The histopathological diagnosis after surgical resection or biopsy served as the reference standard. Given the high aggressiveness and thus lethality of PDAC [33], follow-up data (CT, MRI) > 6 month were used in order to strengthen the clinical diagnosis in cases, where histological confirmation in primary setting was not available. Post-surgery patients were screened for possible recurrence using CT, physical examinations and lab parameters (Carbohydrate-Antigen 19–9 (CA 19–9), C-reactive protein, lipase, amylase) for at least 18 months. Lesions of these patients were classified as recurrences or reactive changes according to previously published criteria [24], in short: In CT, space-occupying, progredient lesions with narrowing of surrounding vessels were considered typical for local recurrences, atrophy of the pancreas over time was considered as typical sign of chronic inflammation and masses in areas of surgery without growth, compression or infiltration of surrounding structures were considered typical for reactive tissue. Static imaging data of the patients included in our dataset has in part been included in previous publications of our group [10, 24, 25].

### ^68^Ga-FAPI-PET/CT imaging

Synthesis and labeling of ^68^Ga-FAPI-46 and was conducted as previously described [34–36]. For PET imaging, a Siemens Biograph mCT Flow scanner was used, according to previously published protocols [11]. Dynamic PET data acquisition was performed covering the upper abdomen and comprised of a series of 28 time frames starting directly after tracer injection continuing until 60 min. The frame duration increased progressively from 30 s to 10 min (min), resulting in a total scanning time of 60 min. After dynamic PET, a wholebody PET acquisition from skull to mid-thigh was started. Images of the PET dynamic series were reconstructed iteratively using OSEM 3D algorithm (matrix: 400 × 400) with 5 iterations and 21 subsets including point spread function (PSF) and time-of-flight (TOF). The reconstruction of wholebody PET images was performed using OSEM 3D algorithm (matrix 200 × 200) with 4 iterations and 8 subsets followed by a Gauss filtering with 5.5 mm filter width. For all patients dynamic and static PET-scans were acquired directly after tracer injection and 60 min (min.) post injection (p. i.) of ^68^Ga-FAPI-46.

### Image evaluation and parametric mapping

PET images were analyzed using PMOD V4.4 (PMOD Technologies LLC, Fällanden, Switzerland) base-licence (PBAS) tool. If necessary, a frame-by-frame motion correction of the PET dynamic images was performed. The analyst was blinded to the type of pancreatic lesion. Volumes of interest (VOIs) were delineated as followed: to assess whole blood input VOIs were placed in the abdominal aorta on eleven adjacent transaxial slices in the first frames of the dynamic PET without partial volume correction or spill-over correction due to sufficient VOI-volume. VOIs of pancreatic lesions were defined on last frames using isocontours at 30–70% of the image maximum according to previously published data of our group [10, 24, 37] in order to achieve optimal alignment between tracer-uptake and the CT-morphological lesion. Additionally, a spherical VOI was placed into the liver. Time-activity-curves (TACs) of these VOIs were created for further analyses in PMOD’s pixel-wise modelling (PXMOD) tool. Compartment modelling (one- (1TC) and two- (2TC) tissue compartment model) of TACs of the liver and pancreatic lesions was performed using the aortic TAC as blood input function in order to calculate parametric maps of the volume of distribution (VD). For 2TC, the ratio K1/k2 was fixed to 0.5 in order to achieve reliable model fits. When using the graphical Logan Plot, the t* value was varied in that way that the fit was performed on last 8 frames (20’ to 60’) of the dynamic PET. In comparison to compartment models output, the graphical analysis Logan plot (LP) was applied to calculate parametric VD maps. LP uses linear regression for analyzing the receptor binding character of a ligand [38]. The same VOIs previously delineated on dynamic PET data were used on static wholebody PET in order to calculate standard uptake values (SUV). Receiver-operating-characteristic (ROC) curves of all signal intensities for the differentiation of PDAC/ILP or recurrent PDAC/PRT were generated.

### Statistical analysis

Descriptive statistical analysis was performed for patients’ characteristics. IBM SPSS Statistics V29 (IBM Deutschland GmbH, Böblingen, Germany) was used to detect outliers from normal distributions applying Grubbs’ criterion: The normally distributed values were z transformed. SPSS showed outliers marked with circles. The z values of these outliers were compared to the Grubbs critical z values at alpha = 0.05. Outliers were classified as true if their z value exceeded the critical Grubbs z. For comparison of static and parametric imaging parameters, unpaired t-tests using Graph Pad Prism 10.2.3 was applied. P-values < 0.05 were considered as significant. Due to the exploratory nature of this analysis, no correction for multiple testing was applied. Receiver operating characteristics (ROC) curve analyses were performed to evaluate the diagnostic accuracy and discriminative power of each parameter. Area under the curve (AUC) were calculated as a global measure of separation between the PDAC and ILP as well as PDAC-recurrence and PRT groups, respectively. A diagnostic threshold representative of our cohort was determined using the ROC curve by identifying the coordinates that yielded the highest combined sensitivity and specificity. This point corresponds to the maximum likelihood ratio (LR+) and ensures the highest discriminatory power for classification. Point estimates for AUC, sensitivity, and specificity were accompanied by their respective 95% confidence intervals (95-CI). Significance was established in instances where the lower bound of the interval remained strictly above the 0.5 threshold.

## Results

### Patient characteristics

**Fig. 1:**
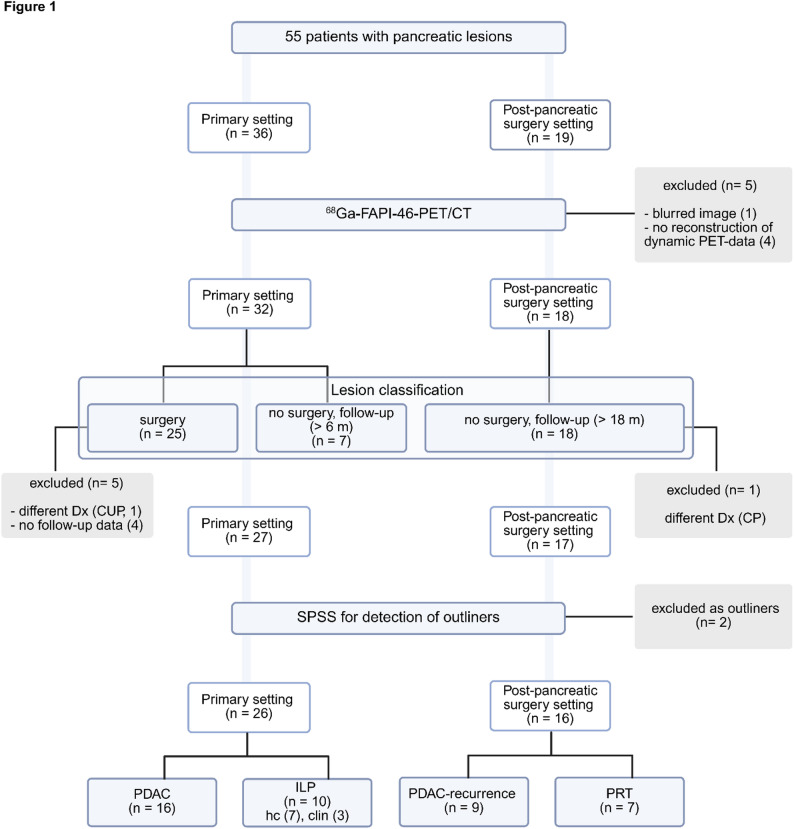
Inclusion process of 55 patients with pancreatic lesions in primary and post-pancreatic surgery setting who were examined by positron emission tomography/ computed tomography with ^68^Gallium-labeled fibroblast activation protein-46 inhibitor (^68^Ga-FAPI-46-PET/CT) including the grounds for exclusion (gray background) at each decision-making level (blue background). Reasons for exclusion were technical at first step (PET-imaging), clinical at second step (lesion classification), e.g. different diagnosis (Dx) like cancer of unknown primary (CUP) and chronic pancreatitis (CP), as well as statistical (IBM SPSS Statistics V29 (IBM Deutschland GmbH, Germany)) at third step. 42 patients were included in the final analysis: In primary setting, 16 patients with pancreatic ductal adenocarcinoma (PDAC) and 10 patients with inflammatory lesions of the pancreas (ILP), 7 histological confirmed (hc) and 3 clinically (clin) strengthened by follow up > 6 months (m). In post-pancreatic surgery setting, 9 patients had PDAC-recurrence and 7 patients showed post-surgery reactive tissue (PRT) strengthened by follow-up > 18 months, respectively

55 patients (36 in primary setting, 19 in post-surgery setting) were examined with ^68^Ga-FAPI-PET/CT. 5 patients were excluded of further analysis due to deficient PET data sets or missing reconstruction of dynamic data, 2 patients were excluded due to different diagnosis after surgery or during follow up, 4 patients were excluded due to missing follow-up data and 2 patients were marked as outliers in statistical analysis with SPSS. Of the included 42 patients (10 female (f), 32 male (m), mean age 68.4 ± 7.2 years (f), 62.5 ± 11.7 years (m)), 26 patients had a primary pancreatic lesion and 16 patients were status post-surgery. Of the primary cases, 16 patients had histologically confirmed (hc) PDAC, 7 patients hc ILP and 3 patients clinically diagnosed ILP. In post-pancreatic surgery setting, 9 patients showed PDAC-recurrence and 7 patients PRT. The specific inclusion process, including the grounds for exclusion at each decision-making level, is illustrated in Fig. 1. Supplemental Table 1 provides a patientwise overview of patient characteristics and diagnoses.

### Differentiation of PDAC and primary inflammatory lesions of the pancreas

**Fig. 2:**
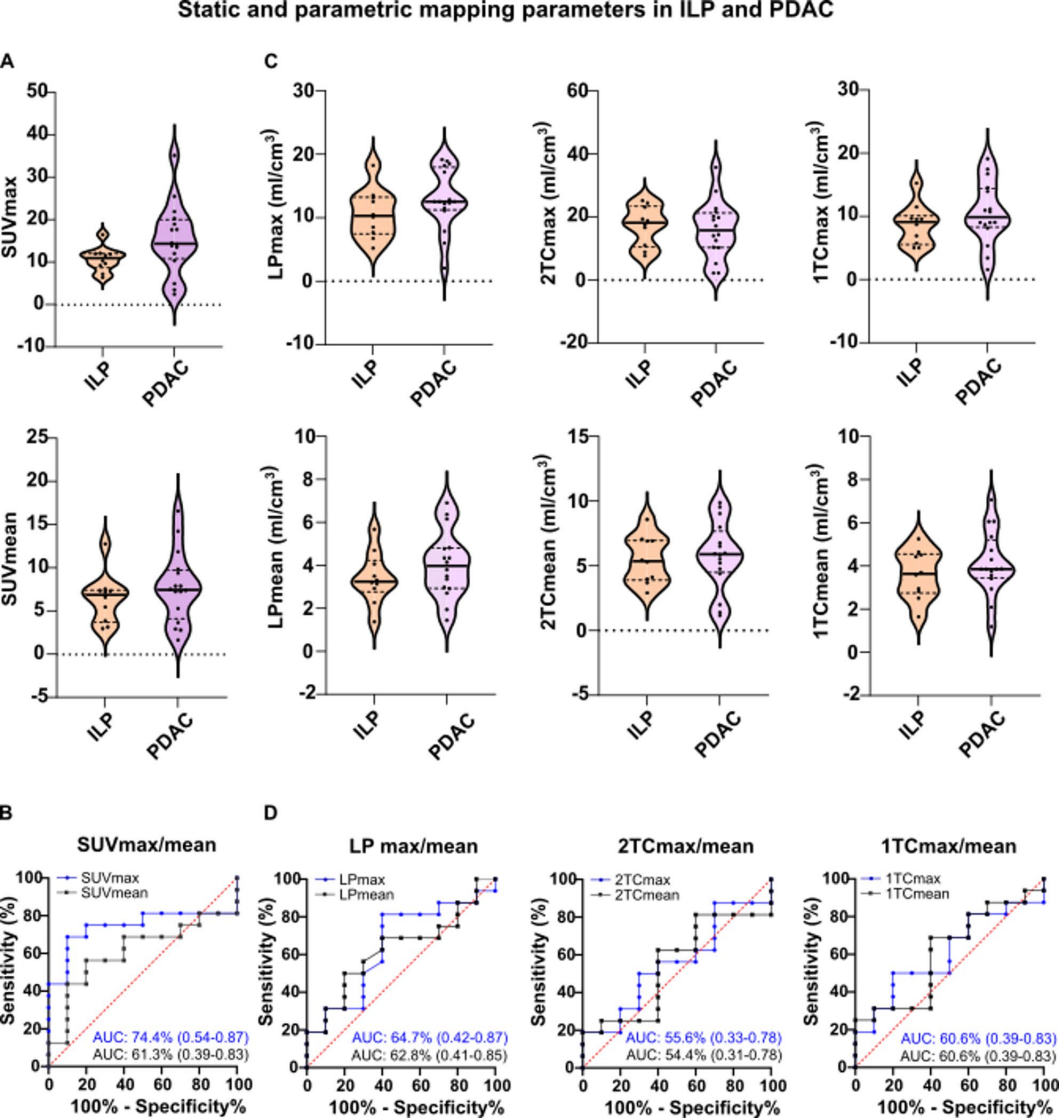
Analysis of static and dynamic ^68^Ga-FAPI-46-PET/CT acquisition with parametric mapping in 16 patients with PDAC and 10 with ILP.** A** SUVmax/mean of primary inflammatory lesions of the pancreas (ILP) and pancreatic ductal adenocarcinomas (PDAC) **B** Area under curve (AUC) with 95%-confidence intervals (95-CI, in parentheses) of SUVmax/mean for distinguishing between ILP and PDAC **C** Maximum and mean signal intensities of ILP and PDAC in Logan plot (LPmax/mean), 2-tissue compartment (2TCmax/mean) and 1-tissue compartment (1TCmax/mean) in milliliter per cubic centimeter (ml/cm^3^). **D** AUC with 95-CI of LP, 2TC and 1TC for distinguishing between PDAC and ILP. No significant differences in signal intensities of ILP and PDAC were observed

In comparison of PDAC and ILP, the mean SUVmax/mean of PDAC (15.39 ± 8.35/ 4.74 ± 2.15) was higher than the mean SUVmax/mean of ILP (10.66 ± 2.86/ 3.72 ± 1.17), but no significant difference was detected (Fig. 2A). With AUCs of SUVmax/mean 74.4% (95-CI: 0.54–0.94)/61.3% (95-CI: 0.39–0.83) the differentiation between PDAC and ILP with static imaging showed moderate sensitivity and specificity (Fig. 2B). The analysis of mean LPmax/mean of PDAC (12.85 ± 4.82 ml/cm^3^/ 4.03 ± 1.54 ml/cm^3^) vs. ILP (10.63 ± 3.86 ml/cm^3^/ 3.41 ± 1.20 ml/cm^3^), mean 1TCmax/mean of PDAC (10.56 ± 4.92 ml/cm^3^/ 4.12 ± 1.49 ml/cm^3^) vs. ILP (8.69 ± 3.20 ml/cm^3^/ 3.60 ± 1.12 ml/cm^3^) and mean 2TCmax/mean of PDAC (16.00 ± 9.07 ml/cm^3^/ 5.74 ± 2.66 ml/cm^3^) vs. ILP (17.27 ± 6.29 ml/cm^3^/ 5.49 ± 1.83 ml/cm^3^) showed non-significantly higher signal intensities in PDAC compared to ILP in LP- and 1TC-, but higher signal intensities in ILP in 2TC-maps (Fig. 2C). Concordantly the AUC of LPmax/mean (64.7%, 95-CI: 0.42–0.87/62.8%, 95-CI: 0.41–0.85), 1TCmax/mean (60.6%, 95-CI: 0.39–0.83/60.6%, 95-CI: 0.38–0.83) and 2TCmax/mean (55.6%, 95-CI: 0.33–0.78/54.4%, 95-CI: 0.31–0.78) showed a low discriminatory power of these parameters (Fig. 2D). The AUC values with 95-CI, the diagnostic thresholds, and the sensitivity and specificity (each with 95-CI) for each parameter are listed in supplementary Table 2.

### Differentiation of PDAC-recurrence and postoperative reactive tissue

**Fig. 3:**
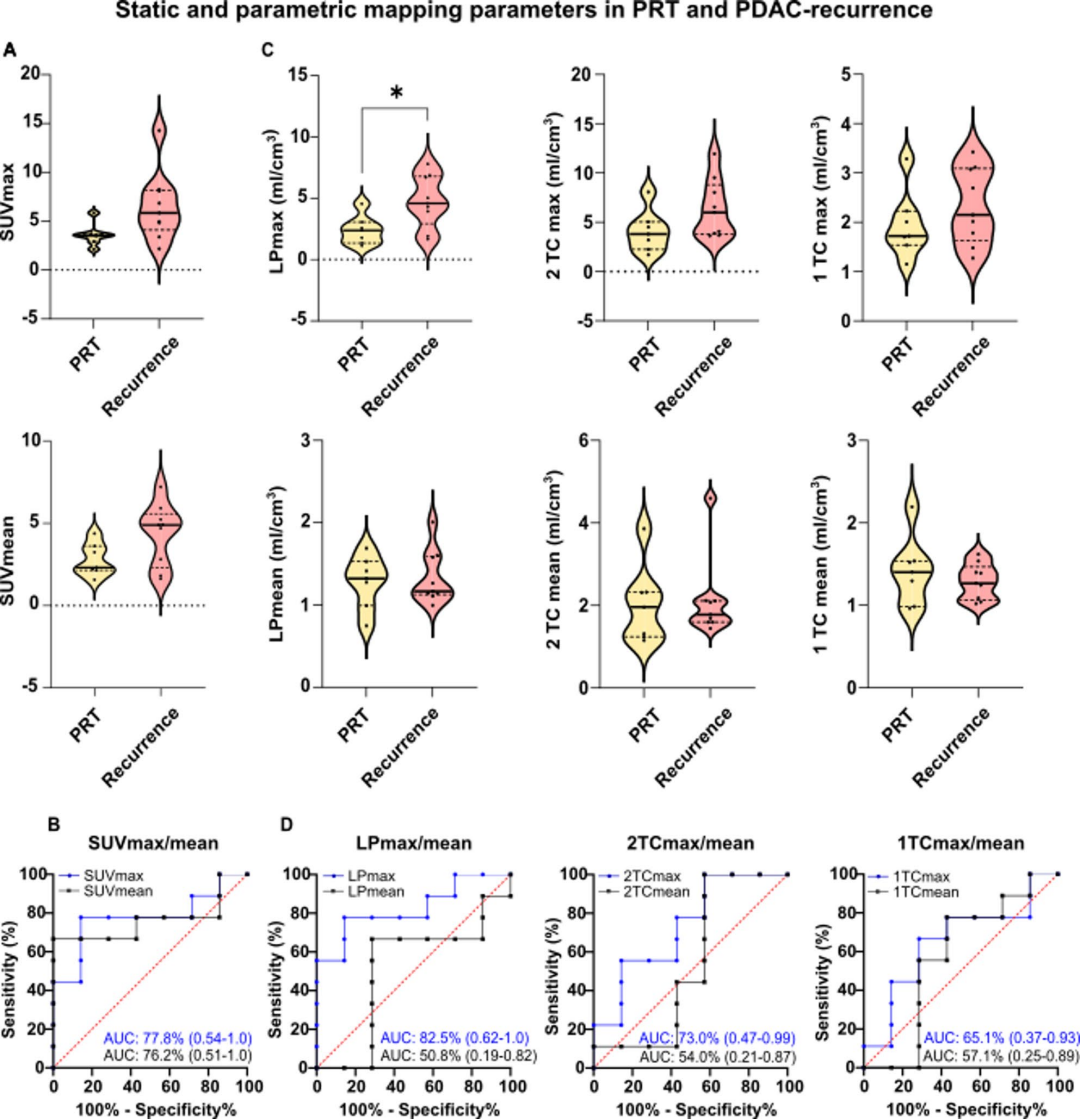
Analysis of static and dynamic ^68^Ga-FAPI-46-PET/CT acquisition with parametric imaging in 7 patients with PRT and 9 with PDAC-recurrence. **A** SUVmax/mean of post-surgery reactive tissue (PRT) and recurrences of pancreatic ductal adenocarcinomas (PDAC) **B** (AUC) with 95%-confidence intervals (95-CI, in parentheses) of SUVmax/mean for distinguishing between PDAC-recurrences (R) and PRT **C** Maximum and mean signal intensities of PRT and PDAC-recurrences in Logan plot (LPmax/mean), 2-tissue compartment (2TCmax/mean) and 1-tissue compartment (1TCmax/mean) in milliliter per cubic centimeter (ml/cm^3^). **D** AUC with 95-CI of LP, 2TC and 1TC for distinguishing between PRT and PDAC-recurrences. * = *p* < 0.05. Besides LPmax, no significant differences in signal intensities of PRT and PDAC-recurrence were observed

Mean SUVmax/mean of PDAC-recurrence (6.52 ± 3.54/ 2.32 ± 0.58) were not significantly higher than of PRT (3.60 ± 1.14/ 1.51 ± 0.37) with moderate AUC of 77.8% (95-CI: 0.54-1.0) /76.2% (95-CI: 0.51-1.0) (SUVmax/mean) (Fig. 3A, B). LP maps showed averagely significantly higher maximum signal intensities in recurrences compared to PRT (4.75 ± 2.12 ml/cm^3^ vs. 2.40 ± 1.17 ml/cm^3^, *p* = 0.02) whereas no significant differences were observed in LPmean (1.33 ± 0.33 ml/cm^3^ vs. 1.28 ± 0.32 ml/cm^3^). Mean 1TCmax/mean of recurrences (2.34 ± 0.77 ml/cm^3^/ 1.29 ± 0.21 ml/cm^3^) vs. PRT (1.95 ± 0.68 ml/cm^3^/ 1.41 ± 0.42 ml/cm^3^) and mean 2TCmax/mean of recurrences (6.37 ± 2.96 ml/cm^3^/ 2.11 ± 0.96 ml/cm^3^) vs. PRT (4.09 ± 2.12 ml/cm^3^/ 2.02 ± 0.95 ml/cm^3^) showed non significantly higher signal intensities in PDAC-recurrence compared to PRT (Fig. 3C). Accordingly, the AUC of LPmax (82.5%, 95-CI: 0.62-1.0) showed a higher discriminatory power than of LPmean (50.8%, 95-CI: 0.19–0.82), 1TCmax/mean (65.1%, 95-CI: 0.37–0.93/57.1%, 95-CI: 0.25–0.89) and 2TCmax/mean (73.0%, 95-CI: 0.47–0.99/54.0%, 95-CI: 0.21–0.87) (Fig. 3D). The AUC values with 95-CI, the diagnostic thresholds, and the sensitivity and specificity (each with 95-CI) for each parameter are listed in supplementary Table 2.

### Visual contrasts and tumor to background ratios for static and parametric imaging

**Fig. 4:**
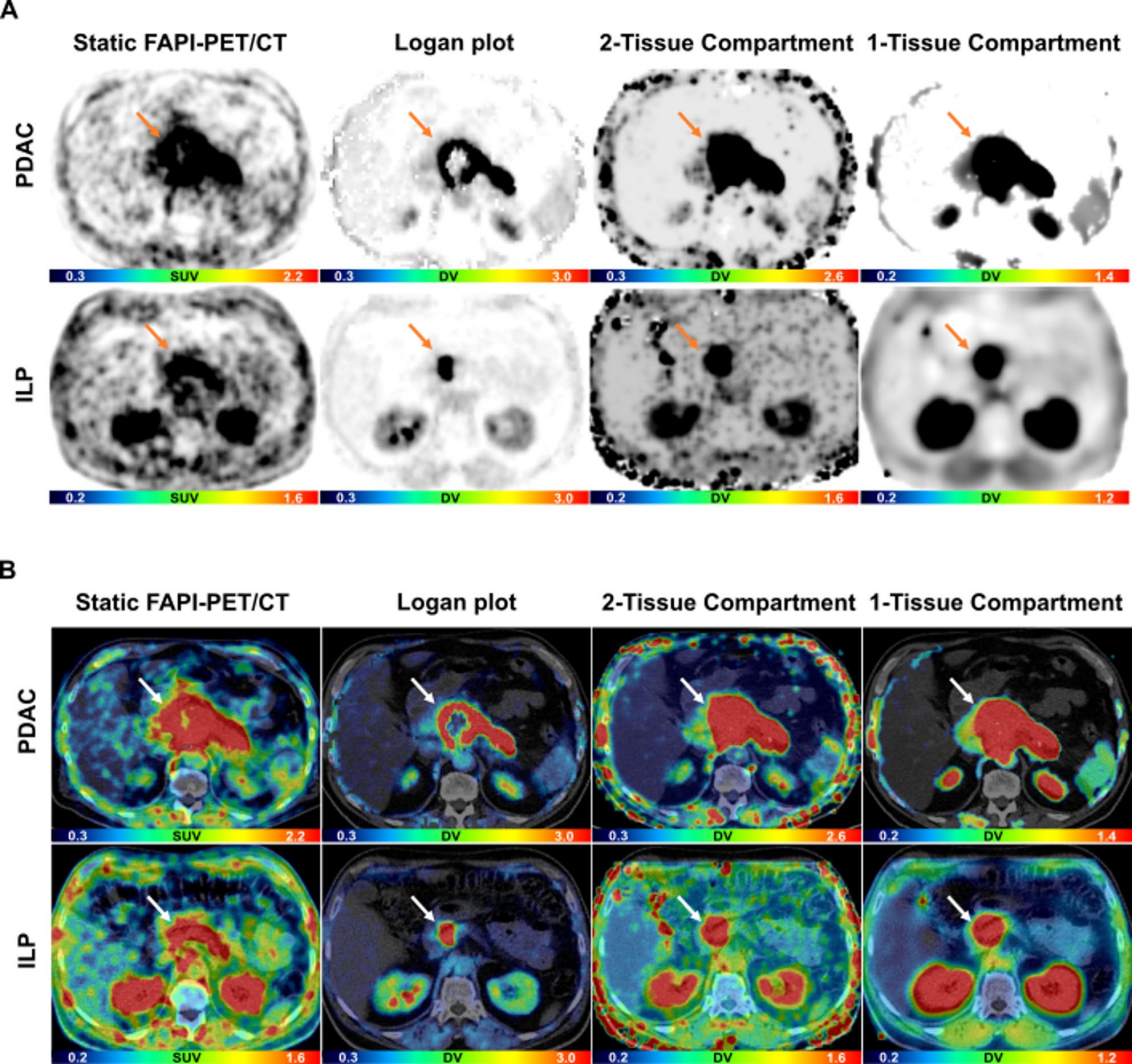
Representative static and parametric imaging data with ^68^Ga-FAPI-46-PET/CT of one patient with PDAC and one patient with ILP. **A** PET imaging data with ^68^Gallium- (^68^Ga) labeled fibroblast activation protein inhibitor-46 (FAPI-46) of a patient with a primary inflammatory lesion of the pancreas (ILP) and a patient with pancreatic ductal adenocarcinoma (PDAC). **B** Corresponding fused PET/CT imaging data. SUV = standardized uptake value (unit less), DV = distribution volume (ml/cm^3^). Arrows indicate PDAC and ILP, respectively

**Fig. 5:**
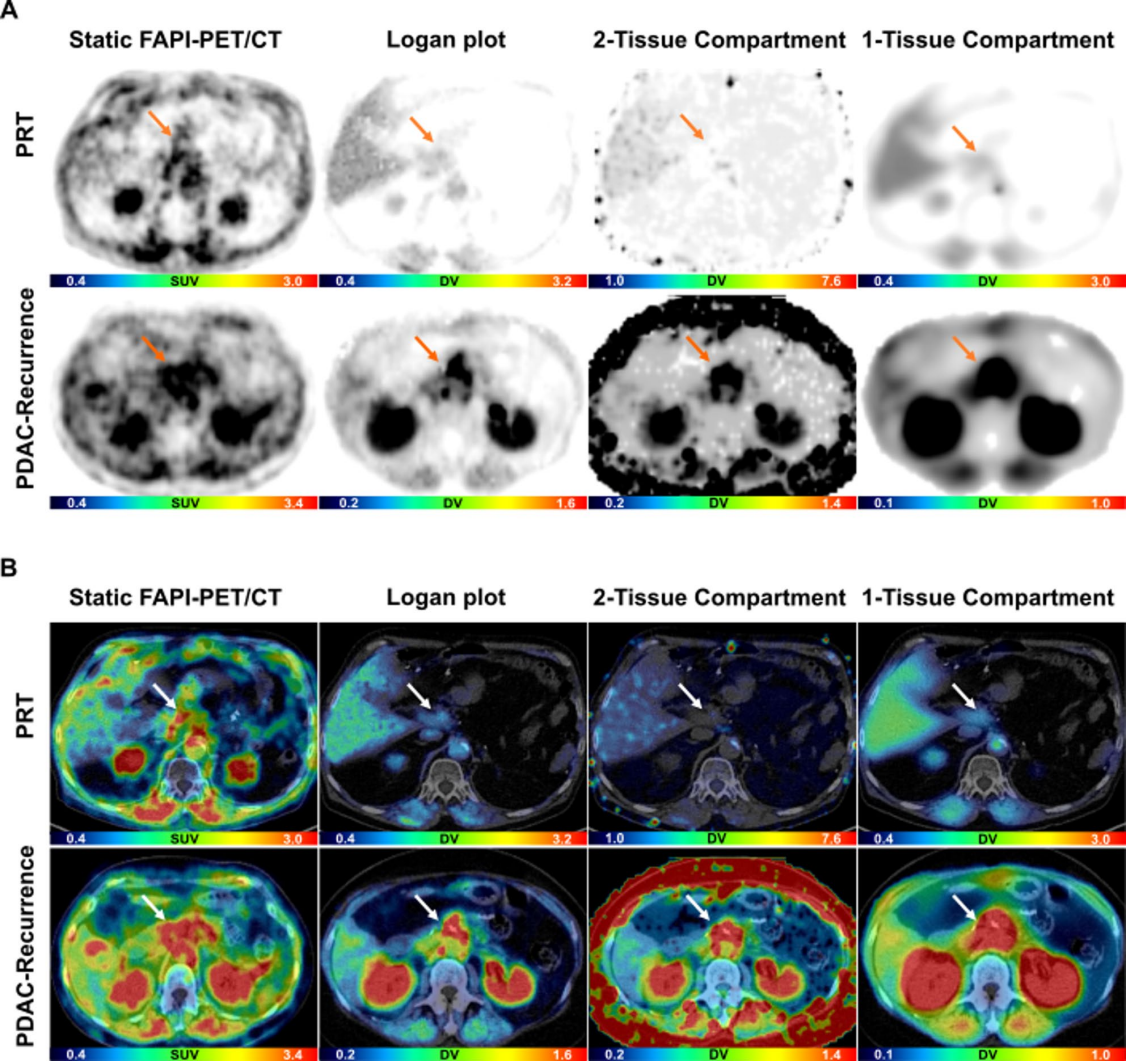
Representative static and parametric imaging data with ^68^Ga-FAPI-46-PET/CT of one patient with PR and one patient with PDAC-Recurrence. **A** PET imaging data with ^68^Gallium- (^68^Ga) labeled fibroblast activation protein inhibitor-46 (FAPI-46) of a patient with post-pancreatic surgery reactive tissue (PRT) and patient with recurrence of a pancreatic ductal adenocarcinoma (PDAC). **B** Corresponding fused PET/CT imaging data. SUV = standardized uptake value (unit less), DV = distribution volume (ml/cm^3^). Arrows indicate PRT and PDAC-Recurrence, respectively

In all cases, parametric maps showed visually higher contrast of the lesions to the surrounding tissues and less background noise in comparison to static imaging. Comparing the maps, imaging with LP achieved visually the highest contrast, while 2TC maps showed the most background noise (Figs. 4 and 5). For parametric imaging, LP-TBRs to fat (*p* < 0.05), stomach (*p* < 0.05) and duodenum (*p* < 0.01) and 2TC-TBRs to fat (*p* < 0.05) and duodenum (*p* < 0.05) showed significant differences for PRT vs. PDAC-recurrence (supplemental Table 2). For static imaging, TBRs showed significant differences for PRT vs. PDAC-recurrence (fat: *p* < 0.05, stomach: *p* < 0.01, duodenum: *p* < 0.05) and for ILP vs. PDAC (fat, *p* < 0.05). There were no significant differences in TBRs to liver for both scenarios (ILP vs. PDAC; PRT vs. recurrence) and to normal pancreas for ILP vs. PDAC due to only sporadic definable healthy pancreatic tissue (supplemental Tables 3 and 4).

### Correlation analysis of static and parametric signal values

Pearson correlation analysis revealed strong positive correlations between all static and parametric maximum signal intensity values (supplemental Table 5).

## Discussion

In our retrospective study, we used parametric mapping derived from dynamic ^68^Ga-FAPI-PET-data in 42 patients with pancreatic lesions in primary (PDAC, ILP) and post-pancreatic surgery setting (PDAC-recurrence, post-surgical reactive tissue) in order to analyze their FAPI-uptake behavior and to specify whether parametric mapping parameters (LP, 2TC, 1TC) allow better differentiation of the lesions in each setting. In primary setting, no significant difference in signal intensities neither in static nor in parametric imaging parameters were observed. With regard to the differentiation of PDAC-recurrences versus postoperative tissue, LPmax maps showed significant differences, while static and other parametric mapping parameters yielded no statistically significant results, highlighting the complexity of their clinical differentiation. The comparison of analyzed maps revealed high contrast in LP- and 1TC- while 2TC-maps showed more background noise.

The analysis of parametric imaging with FAPI-PET data was performed only once prior this study by Chen et al. investigating the kinetics of FAPI-04 in dynamic total-body data in patients with pancreatic and gastric cancer. Comparing reversible 1TC-, irreversible and reversible 2TC- and LP-maps, they depicted 2TC-maps as most appropriate parametric model for ^68^Ga-FAPI-04 [30]. Dimitrakopoulou-Strauss et al. described in their analysis of parametric imaging with ^18^F-FDG that 2TC-maps show more background noise while regression images lead to high contrast to the surrounding tissues, which can allow better image reading and thus tumor delineation. This can be advantageous especially for tumor lesions with enhanced tracer uptake localized in organs with physiologically high tracer uptake such as liver. For tumor lesions with a low FDG-uptake like lung metastasis localized in an organ with a physiologically low tracer uptake no better results were achieved with parametric imaging in comparison to SUV images [32].

Distinguishing elevated FAPI-uptake remains challenging in clinical practice as both malignant tumors and severely inflamed tissue overexpress FAP leading to high uptake values [37, 39–41]. This is driven by similar biological mechanisms. In both inflammation and malignancy, fibroblast populations undergo a fundamental shift, expanding and acquiring pathological traits that sustain disease. The core similarity lies in their functional reprogramming: just as shared signals across organs like the lung and liver trigger myofibroblast gene programs, diverse CAFs promote tumor progression. Both cell types act as primary architects of the diseased microenvironment by stimulating angiogenesis and aggressively remodeling the extracellular matrix. The immunological role is another shared hallmark. In both contexts, fibroblasts function as active participants in leukocyte recruitment and immune modulation. In chronic inflammation, fibroblasts adopt a phenotype reminiscent of fibroblastic reticular cells which are present in lymphoid organs thereby promoting sustained recruitment and retention of immune cells. While lymphoid tissues usually return to homeostasis after an infection clears, fibroblasts in chronic inflammation keep retaining lymphocytes. This mechanism is mirrored by CAFs, which orchestrate the immune landscape to facilitate metastasis and establish an immunosuppressive milieu which shields tumors from immunosurveillance [42–45].

As our study demonstrates, this complexity, which is reflected by FAPI-uptake, persists not only in static but also in dynamic FAPI-PET imaging, particularly within the pancreas. Various methodological approaches have been developed to analyze dynamic PET data to distinguish between benign and malignant pathologies [10, 46, 47]. A promising framework in view of differentiation of morphologically unclear pancreatic lesions was recently introduced by Röhrich et al. (2026), involving the application of “digital biopsies” to benign and malignant pancreatic lesions. In this approach, voxels were standardized by size to reflect the distinct kinetic profiles of PDAC, ILP, PDAC recurrence, and PRT. The results indicated that clusters representing pathological pancreatic lesions were clearly demarcated from healthy control tissues. Furthermore, this distinctiveness was observed within the pathology groups: although a degree of visual overlap persisted in both primary and postoperative settings, the implementation of combined networks yielded visually discernible clusters for the majority of malignant and benign lesions, respectively [47]. Despite the relatively small number of histologically confirmed lesions, especially of the PRT in follow-up setting, digital biopsy seems to be a more precise tool than parametric mapping for this highly complex clinical constellation. Although the incremental diagnostic value of dynamic FAPI-PET imaging is not fully explored to date and the preferable method of data processing and analysis requires further investigation, this acquisition method provides extensive, potentially important additional datasets [10, 47–49] compared to static imaging alone and should therefore be implemented into clinical routine provided that workflows permit it.

Several limitations of this study must be considered. The major limitation is the relatively small number of patients, especially per group, which can underpower the ability to detect statistically significant differences between these groups. As smaller studies are more susceptible to type II errors this might has contributed to the mostly non-significant findings. Moreover, because no adjustment for multiple comparisons was applied, the likelihood of false-positive findings is elevated, so reported relationships should be interpreted with appropriate caution. In addition, due to the small sample size, we were unable to perform detailed subgroup analysis, which could have revealed effects on FAPI-uptake such as PDAC grading or previous therapy vs. therapy naivety. In this context, it has to be taken into account that our study is single-center with patients treated in a highly specialized pancreatic center, which may have led to referral bias. As patients partially receive neoadjuvant chemotherapy at the university hospital of Heidelberg, this may influence the number of active fibroblasts in a primary as well as recurrent and post-surgery situation and thus the FAPI-uptake behavior of the observed lesions. Lastly, the inclusion of patients without histological confirmation may lead to a misclassification bias by attributing the observed FAPI-uptakes to a probably histopathological wrong group. At this point, however, given the high aggressiveness and associated lethality of PDAC [33], it is important to note the relatively long follow-up period of > 6 months for primary cases and 18 months for patients in the postoperative course: In the absence of lesion growth or patient mortality, it can be assumed with relative certainty, both clinically and radiomorphologically, that the lesion is of non-malignant origin. Multicenter studies with a larger patient cohort, histological confirmations and subgroup analysis are thus needed to gain more robust findings than our pilot analysis.

## Conclusion

Parametric mapping of dynamic FAPI-PET data represents a new area of research. In our analysis, LPmax showed a significant difference in the post-pancreatic surgery setting, while other parameters of parametric mapping did not yield statistically significant results in the primary and postoperative setting, suggesting that the inclusion of LP maps may offer additional value in distinguishing between PDAC-recurrence and PRT. However, given the limited sample size, these results should be interpreted with caution. Future studies with larger cohorts, as well as alternative approaches to analyzing dynamic imaging data, are needed.

## Supplementary Information

Below is the link to the electronic supplementary material.

Supplementary Material 1

## Data Availability

All data that is presented in this manuscript has been acquired and processed in the University hospitals of Heidelberg and Mainz. All data is stored in these centers and is avaiable from the corresponding author in reasonable request.
