# Supplementary material for: Parametric mapping of dynamic 68Ga FAPI-46-PET data of 42 patients with pancreatic lesions
Source: Cancer Imaging. 2026 Apr 28;26:55. doi: 10.1186/s40644-026-01040-w (PMC13127050; doi:10.1186/s40644-026-01040-w)
Supplement: Supplementary file 1 — Supplementary Material 1 [file 40644_2026_1040_MOESM1_ESM.docx]

**Supplemental material**

**Parametric mapping of dynamic ^68^Ga FAPI-46-PET data of 42 patients with pancreatic lesions**

Anna-Maria Spektor^1^, Isabelle von Goetze^1^, Hans-Georg Buchholz^1^, Ulrike Heger^2^, Matthias Lang^2^, Jakob Liermann^3^, Maximilian Knoll^3^, Klaus Herfarth^3^, Mathias Schreckenberger^1^, Jürgen Debus^3^, Uwe Haberkorn^4^, Manuel Röhrich^1^

**Affiliations:**

1 University Medical Center Mainz, Department of Nuclear Medicine, Mainz, Germany

2 University Hospital Heidelberg, Department of Surgery, Heidelberg, Germany

3 University Hospital Heidelberg, Department of Radiation Oncology, Heidelberg, Germany

4 University Hospital Heidelberg, Department of Nuclear Medicine, Heidelberg, Germany

**Supplemental table 1: Patients´ characteristics**

| **N** | **Sex** | **Age** | **Diagnosis** | **Localization** | **Diagnostic method** | **Group** |
| --- | --- | --- | --- | --- | --- | --- |
| **1** | M | 44 | Necrotic pancreatitis | Head | Histologically/ surgery | **ILP** |
| **2** | M | 66 | Chronic pancreatitis | Head | CT/ clinically |  |
| **3** | M | 60 | PanIN + Pancreatitis | Head | Histologically/ surgery |  |
| **4** | M | 57 | Autoimmune pancreatitis | Head + tail | Histologically/ surgery |  |
| **5** | M | 37 | Chronic pancreatitis | Tail | Histologically/ surgery |  |
| **6** | F | 73 | Pancreatitis | Head | CT/ clinically |  |
| **7** | M | 47 | Suspected pancreatitis |  | CT/ clinically |  |
| **8** | M | 55 | Autoimmune pancreatitis | Head | Histologically/ surgery |  |
| **9** | F | 60 | PanIN + Pancreatitis | Head + Cauda | Histologically/ surgery |  |
| **10** | M | 51 | PanIN + Pancreatitis | Cauda | Histologically/ surgery |  |
| **11** | M | 78 | High grade IPMN | Head + Cauda | Surgery | **PDAC** |
| **12** | M | 77 | PDAC | Cauda |  |  |
| **13** | F | 67 | PDAC, G2 | Corpus |  |  |
| **14** | M | 73 | High grade IPMN | Head |  |  |
| **15** | M | 37 | PDAC, G3 | Head |  |  |
| **16** | M | 67 | PDAC | Head |  |  |
| **17** | M | 72 | PDAC | Head |  |  |
| **18** | M | 61 | PDAC | Head + Corpus |  |  |
| **19** | M | 75 | IPMN with associated PDAC | Corpus + Cauda |  |  |
| **20** | M | 72 | PDAC, G4 | Head |  |  |
| **21** | M | 64 | PDAC, G1 | Head |  |  |
| **22** | M | 68 | PDAC, G1 | Cauda |  |  |
| **23** | M | 56 | PDAC, G2 | Cauda |  |  |
| **24** | F | 73 | PDAC, G2 | Head + Corpus |  |  |
| **25** | F | 69 | PDAC | Head |  |  |
| **26** | M | 67 | PDAC | Tail |  |  |
| **27** | M | 49 | Reactive tissue | Head | CT/ clinically | **PRT** |
| **28** | M | 53 |  | Cauda |  |  |
| **29** | M | 66 |  | Head + Cauda |  |  |
| **30** | M | 73 |  | Head |  |  |
| **31** | M | 59 |  | Head |  |  |
| **32** | M | 49 |  | Head |  |  |
| **33** | M | 71 |  | Head |  |  |
| **34** | F | 62 | PDAC-recurrence | Head |  | **PDAC-Recurrence** |
| **35** | M | 61 |  | Head |  |  |
| **36** | M | 67 |  | Head |  |  |
| **37** | F | 64 |  | Head |  |  |
| **38** | F | 59 |  | Head |  |  |
| **39** | M | 80 |  | Head |  |  |
| **40** | F | 83 |  | Head |  |  |
| **41** | M | 72 |  | Head |  |  |
| **42** | F | 69 |  | Head |  |  |
| Abbreviations: m: male, f: female, PanIN: pancreatic intraepithelial neoplasia, IPMN: intraductal papillary mucinous neoplasm, PDAC: pancreatic ductal adenocarcinoma, CT: computer tomography, ILP: inflammatory lesions of the pancreas, PRT: postoperative reactive tissue | | | | | | |

**Supplemental table 2**

|  | **Parameter** | **AUC (%)** | **95-CI** | **Diagnostic threshold** | **Sensitivity (%) (95-CI)** | **Specificity (%) (95-CI)** | **LR** |
| --- | --- | --- | --- | --- | --- | --- | --- |
| **ILP/PDAC** | SUVmax | 74.4 | 0.54-0.87 | 12.80 | 68.75  (44.4-85.8) | 90.0  (59.6-99.5) | 6.87 |
|  | SUVmean | 61.3 | 0.39-0.83 | 7.34 | 56.25  (33.2-76.9) | 80.0  (49.0-96.5) | 2.81 |
|  | LPmax | 64.7 | 0.42-0.87 | 10.81 | 81.25  (56.99-93.41) | 60.0  (31.27-83.18) | 2.03 |
|  | LPmean | 62.8 | 0.41-0.85 | 4.11 | 50.0  (28.0-72.0) | 80.0  (49.0-96.5) | 2.50 |
|  | 2TCmax | 55.6 | 0.33-0.78 | 15.53 | 50.0  (28.0-72.0) | 70.0  (39.7-89.2) | 1.67 |
|  | 2TCmean | 54.4 | 0.31-0.78 | 5.48 | 62.50  (38.6-81.5) | 60.0  (31.3-83.2) | 1.56 |
|  | 1TCmax | 60.6 | 0.39-0.83 | 10.20 | 50.0  (28.0-72.0) | 80.0  (49.0-96.5) | 2.50 |
|  | 1TCmean | 60.6 | 0.38-0.83 | 3.72 | 68.75  (44.4-84.8) | 60.0  (31.3-83.2) | 1.72 |
| **PRT/PDAC-recurrence** | SUVmax | 77.8 | 0.54-1.0 | 4.91 | 66.67  (35.42-87.94) | 85.71  (48.69-99.27) | 4.67 |
|  | SUVmean | 76.2 | 0.51-1.0 | 4.50 | 66.67  (35.42-87.94) | 100.0  (64.6-100) |  |
|  | LPmax | 82.5 | 0.62-1.0 | 3.49 | 77.78  (45.26-96.05) | 85.71  (48.69-99.27) | 5.44 |
|  | LPmean | 50.8 | 0.19-0.82 | 1.27 | 66.67  (35.42-87.94) | 71.43  (35.89-94.92) | 2.33 |
|  | 2TCmax | 73.0 | 0.47-0.99 | 5.53 | 55.56  (26.67-81.12) | 85.71  (48.69-99.27) | 3.89 |
|  | 2TCmean | 54.0 | 0.21-0.87 | 1.52 | 88.89  (56.50-99.43) | 42.86  (15.82-74.95) | 1.56 |
|  | 1TCmax | 65.1 | 0.37-0.93 | 2.46 | 44.4  (18.89-73.33) | 85.71  (48.69-99.27) | 3.11 |
|  | 1TCmean | 57.1 | 0.25-0.89 | 1.40 | 77.78  (45.26-96.05) | 57.14  (25.05-84.18) | 1.82 |
| Abbreviations: PDAC: pancreatic ductal adenocarcinoma; ILP: inflammatory lesions of the pancreas; PRT: postoperative reactive tissue; SUV: standardized uptake values; LP: logan plot; 2TC: 2 tissue compartment; 1TC: 1 tissue compartment; max: maximum; AUC: area under curve; 95-CI: 95%-confidence interval; LR: likelihood ratio | | | | | | | |

**Supplemental table 3: PRT vs. PDAC-recurrence**

| **Background** | **Static** | **Logan** | **2-tissue** | **1-tissue** |
| --- | --- | --- | --- | --- |
| **Liver** | 0.2387 | 0.0738 | 0.0867 | 0.7201 |
| **Fat** | **0.0120** | **0.0151** | **0.0432** | 0.4355 |
| **Stomach** | **0.0083** | **0.0366** | 0.1002 | 0.2612 |
| **Duodenum** | **0.0152** | **0.0081** | **0.0360** | 0.2049 |

**Supplemental table 4: ILP vs. PDAC**

| **Background** | **Static** | **Logan** | **2-tissue** | **1-tissue** |
| --- | --- | --- | --- | --- |
| **Liver** | 0.1654 | 0.4487 | 0.3394 | 0.6271 |
| **Fat** | **0.0467** | 0.0950 | 0.7285 | 0.1163 |
| **Stomach** | 0.0549 | 0.2119 | 0.8723 | 0.1997 |
| **Duodenum** | 0.0619 | 0.1168 | 0.5346 | 0.3812 |
| **Normal pancreas** | 0.1361 | 0.6485 | 0.6012 | 0.1994 |

**Supplemental table 5: Pearson Correlation**

| **Confidence Intervals** | | | | |
| --- | --- | --- | --- | --- |
|  | **Pearson Correlation** | **p-values**  **(2-tailed)** | **95% Confidence Intervals (2-tailed)*** | |
|  |  |  | **Lower** | **Upper** |
| **SUVmax – LP** | 0.701 | < 0.001 | 0.505 | 0.828 |
| **SUVmax – 2TC** | 0.542 | < 0.001 | 0.285 | 0.726 |
| **SUVmax – 1TC** | 0.679 | < 0.001 | 0.472 | 0.814 |
| **2TC - LP** | 0.693 | < 0.001 | 0.494 | 0.824 |
| **1TC – 2TC** | 0.780 | < 0.001 | 0.624 | 0.876 |
| **1TC - LP** | 0.876 | < 0.001 | 0.780 | 0.932 |
| *Estimation is based on Fisher´s r-to-z transformation | | | | |
